# Supplementary material for: One-Step Co-Electrodeposition of Copper Nanoparticles-Chitosan Film-Carbon Nanoparticles-Multiwalled Carbon Nanotubes Composite for Electroanalysis of Indole-3-Acetic Acid and Salicylic Acid
Source: Sensors (Basel). 2022 Jun 13;22(12):4476. doi: 10.3390/s22124476 (PMC9228024; doi:10.3390/s22124476)
Supplement: Supplementary file 1 [file sensors-22-04476-s001.zip › sensors-1713816-supplementary.pdf]

Supplementary Material

One-Step Co-Electrodeposition of Copper Nanoparticles-Chitosan Film-Carbon Nanoparticles-Multiwalled Carbon Nanotubes Composite for Electroanalysis of Indole-3-Acetic Acid and Salicylic Acid

Yiwen Kuang [1], Mengxue Li [2], Shiyu Hu [2], Lu Yang [2], Zhanning Liang [2], Jiaqi Wang [2], Hongmei Jiang [2], Xiaoyun Zhou [1,*] and Zhaohong Su [2,*]

[1] College of Bioscience and Biotechnology, Hunan Agricultural University, Changsha 410128, China; kuangyiwen@stu.hunau.edu.cn

[2] College of Chemistry and Materials Science, Hunan Agricultural University, Changsha 410128, China; limengxue@stu.hunau.edu.cn (M.L.); hushiyu@stu.hunau.edu.cn (S.H.); yanglu00@stu.hunau.edu.cn (L.Y.); liangzhanning@stu.hunau.edu.cn (Z.L.); wangjiaqi@stu.hunau.edu.cn (J.W.); jhmndcn@hunau.edu.cn (H.J.)

* Correspondence: xyzhou71@hunau.edu.cn (X.Z.); zhaohongsu@hunau.edu.cn (Z.S.)

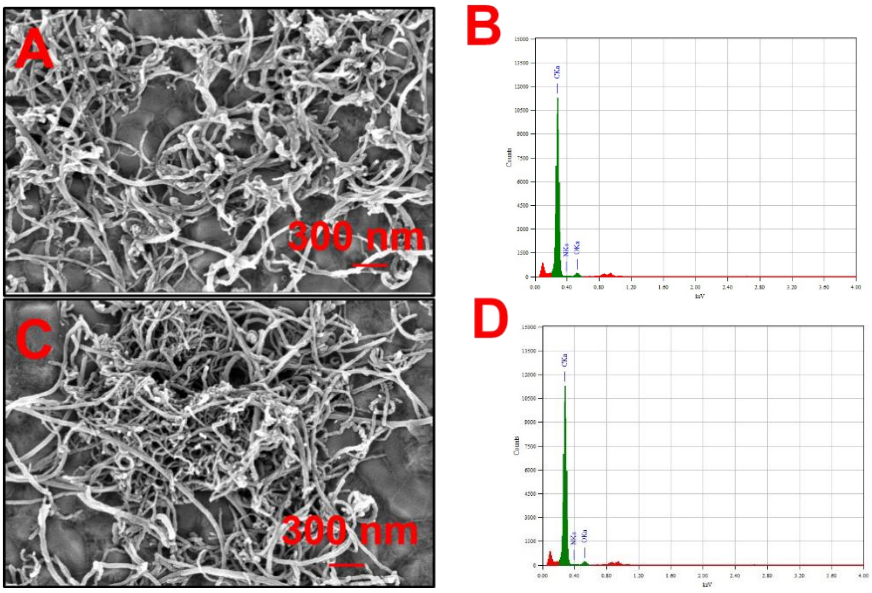

Figure S1. SEM images of MWCNTs/GCE (A) and CNPs-MWCNTs/GCE (C). EDX spectrum of MWCNTs (B) and CNPs-MWCNTs (D).

SEM was used to characterize MWCNTs/GCE and CNPs-MWCNTs/GCE (Figure R5). There is no obvious morphological change between MWCNTs/GCE (Figure R5A) and CNPs-MWCNTs/GCE (Figure R5C), because CNPs are relatively small and cannot be clearly seen in SEM. Figure R5B and Figure R5D are EDX spectrum of MWCNTs and CNPs-MWCNTs. It can be seen that they both have C and O elements, while CNPs-MWCNTs have more N elements than MWCNTs, indicating that CNPs-MWCNTs composites were successfully prepared.

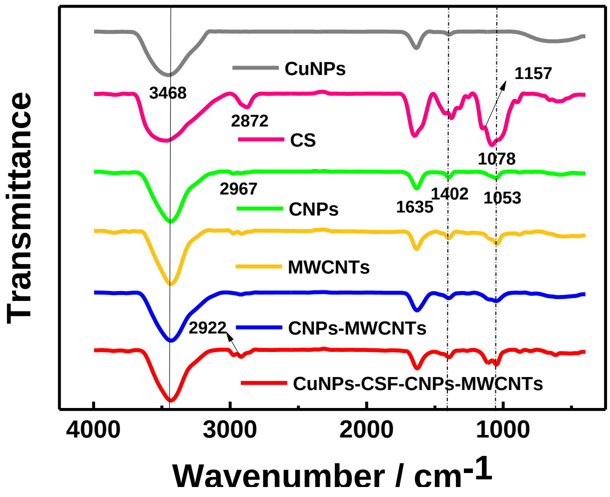

Figure S2. FT-IR spectra of CuNPs, CS, CNPs, MWCNTs, CNPs-MWCNTs, and CuNPs-CSF-CNPs-MWCNTs, respectively.

Figure S2 is FT-IR spectra of CuNPs, CS, CNPs, MWCNTs, CNPs-MWCNTs, and CuNPs-CSF-CNPs-MWCNTs. The peaks at 1402 cm^{-1} and 1053 cm^{-1} correspond to the bending vibration of $-CH_2$ and the stretching vibration of C=O, respectively[1]. Compared with CS, the O-H and N-H stretching vibration absorption peaks of CuNPs-CSF-CNPs-MWCNTs shifted to the wavelet number at 3468 cm^{-1}. The C-H stretching vibration absorption peaks appear at 2967 cm^{-1} and 2922 cm^{-1}, and C-H vibration is related to the disappearance of pyranose at 1157 cm^{-1}. These properties can explain the formation of CSF and the dehydration of the pyranose ring by the chitosan chain [2].

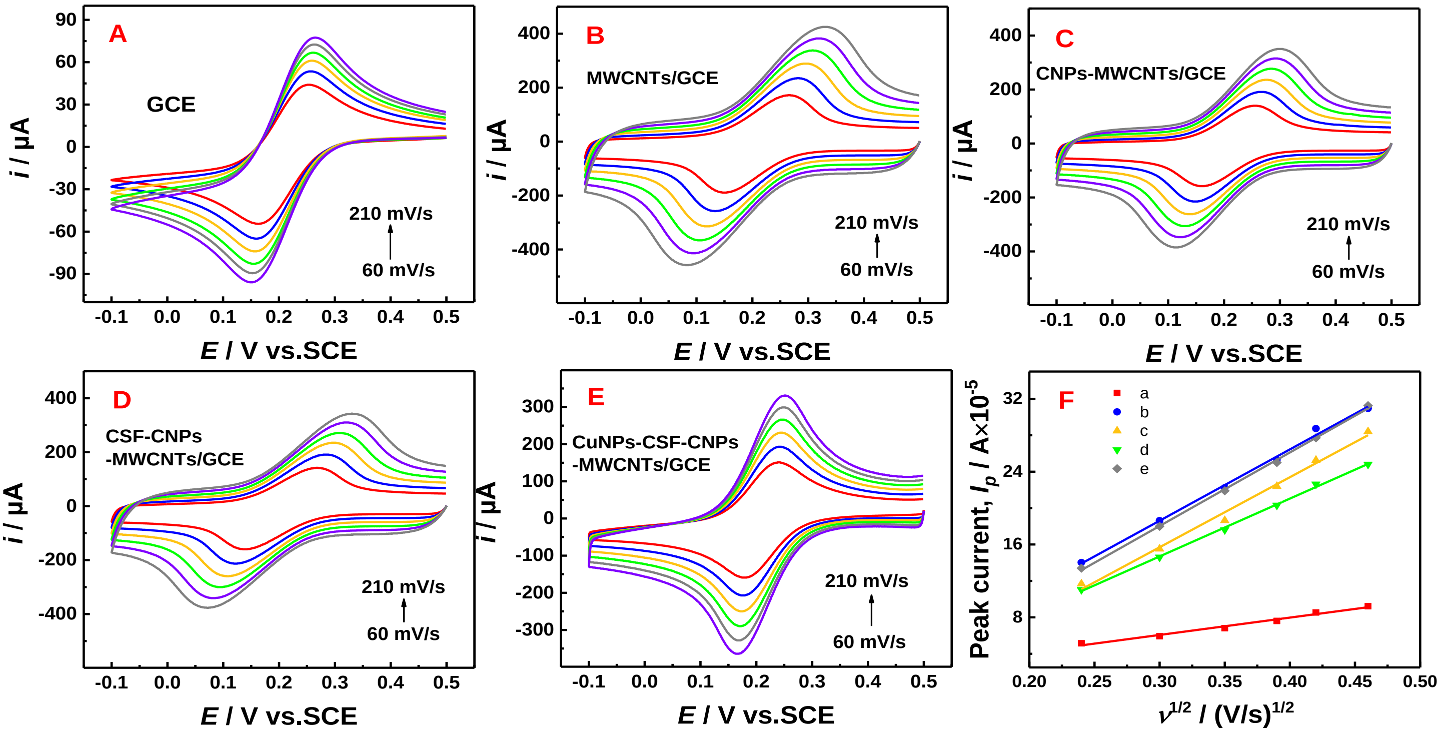

Figure S3. CVs of bare GCE (A), MWCNTs/GCE (B), CNPs-MWCNT/GCE (C), CSF-CNPs-MWCNT/GCE (D), and CuNPs-CSF-CNPs-MWCNTs/GCE (E) measured at different scan rates in 5.0 mM $[Fe(CN)_6]^{3-/4-}$ + 0.5 M KCl. (F) Linear relationship between peak current (I_p) and square root of scan rate ($\nu^{1/2}$) for different modified electrodes (a-e: GCE, MWCNTs/GCE, CNPs-MWCNT/GCE, CSF-CNPs-MWCNT/GCE, CuNPs-CSF-CNPs-MWCNTs/GCE).

The electroactive surface areas of bare GCE (Figure S3A), MWCNTs/GCE (Figure S3B), CNPs-MWCNT/GCE (Figure S3C), CSF-CNPs-MWCNT/GCE (Figure S3D) and CuNPs-CSF-CNPs-MWCNTs/GCE (Figure S3E) were further investigated by CV. Figure S3F shows the linear relationship between the peak current (I_p) and the square root of the scan rate ($\nu^{1/2}$) for different electrodes at different scan rates (0.06 ~ 0.21 V s^{-1}) in 5 mM $[Fe(CN)_6]^{3-/4-}$ solution containing 0.5 M KCl. GCE: $I_p(A) = (1.9 \times 10^{-4})\nu^{1/2}(V/s) + 3.5422\times10^{-6}$ ($R^2 = 0.9845$), MWCNTs/GCE: $I_p(A) = (7.83 \times 10^{-4})\nu^{1/2}(V/s) - 4.8887\times10^{-5}$ ($R^2 = 0.9949$), CNPs-MWCNTs/GCE: $I_p(A) = (7.69 \times 10^{-4})\nu^{1/2}(V/s)$ -

7.3812×10^{-5} (R^2 = 0.9922), CSF-CNPs-MWCNT/GCE: I_p(A) = (6.35×10^{-4})$\nu^{1/2}$(V/s) - 4.363×10^{-5} (R^2 = 0.9894), CuNPs-CSF-CNPs-MWCNTs/GCE: I_p(A) = (8.07×10^{-4})$\nu^{1/2}$(V/s) - 6.1822×10^{-5} (R^2 = 0.9989).

The Randles-Sevcik equation is used to calculate the electroactive surface area of different modified electrodes[3, 4]:

$$I_p=2.69\times10^5 n^{3/2} A D^{1/2} \nu^{1/2} C_0 \quad (1)$$

$$A=B/(2.69\times10^5 n^{3/2} D^{1/2} C_0) \quad (2)$$

where n is the number of transfer electrons, A (cm^2) is the electroactive surface area, D (cm^2/s) is related to the diffusion coefficient, ν (V/s) is the scanning rate, C_0 (mol/cm^3) is the concentration of $[Fe(CN)_6]^{3-/4-}$, and B is the slope of the linear relationship (Figure R1F). When the electrolyte is 5 mM $[Fe(CN)_6]^{3-/4-}$ solution, n = 1, D = 6.3×10^{-6} cm^2/s, from eqn (2), the electroactive surface area of MWCNTs/GCE, CNPs-MWCNTs/GCE, CSF-CNPs-MWCNT/GCE and CuNPs-CSF-CNPs-MWCNTs/GCE can be calculated as 0.233 cm^2, 0.228 cm^2, 0.189 cm^2, 0.240 cm^2, respectively, which is much higher than that of bare GCE (0.056 cm^2).

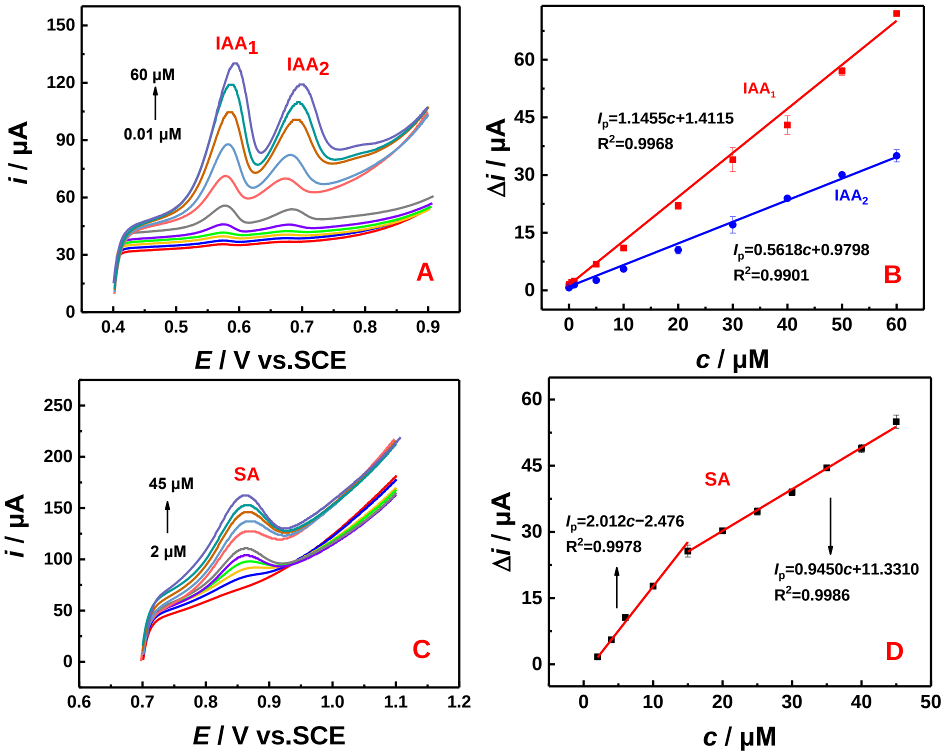

Figure S4. LSV curve of CuNPs-CSF-CNPs-MWCNTs/GCE in 0.1 M PBS (pH=7.0) containing different concentrations of IAA (0.01, 0.1, 0.5, 1, 5 ,10, 20, 30, 40, 50, 60 μM) (A) and SA (2, 4, 6, 10, 15, 20, 25, 30, 35, 40, 45 μM) (C), respectively. Linear correlation curve between peak current and concentrations of IAA_1 (B), IAA_2 (B), and SA (D), respectively.

Figure S4 shows the detection results of IAA (Figure S4A-B) and SA (Figure S4C-D) by CuNPs-CSF-CNPs-MWCNTs/GCE under the optimal experimental conditions. Figure S4A shows the LSV responses of different concentrations of modified electrodes to IAA_1 and IAA_2. Figure S4B shows the linear relationship between the peak current and the concentrations of IAA_1 and IAA_2. A good linear relationship was found for IAA_1 and IAA_2 from 0.01 to 60 μM. The linear regression equations were I_p(μA) = 1.1455c (μmol/L) + 1.4115 (R^2 = 0.9968) and I_p(μA) = 0.5618c (μmol/L) + 0.9798 (R^2 = 0.9901), respectively, and the detection limit was 0.0078 μM and 0.0091 (*S/N* = 3). Figure S4C shows the LSV responses of the modified electrodes at different concentrations to SA. Figure S4D is the linear relationship between peak current and SA concentration. In the 2-45 μM range, there is a good linear relationship between peak current and SA concentration: I_p(μA) = 2.012c (μmol/L) – 2.476, (R^2 = 0.9978) and I_p(μA) = 0.9450c (μmol/L) + 11.3310, (R^2 = 0.9986). The detection limit was 0.24 μM (*S/N* = 3). Since the sensitivity of the first oxidation peak is larger than that of the second oxidation peak (Figure R3B), the first oxidation peak was selected as the research object.

References

1. Ma, B.; Li, X.; Qin, A.; He, C. A comparative study on the chitosan membranes prepared from glycine hydrochloride and acetic acid. *Carbohydr. Polym.* **2013**, *91*, 477-482.
2. Yang, Y.; Cui, J.; Zheng, M.; Hu, C.; Tan, S.; Xiao, Y.; Yang, Q.; Liu, Y. One-step synthesis of amino-functionalized fluorescent carbon nanoparticles by hydrothermal carbonization of chitosan. *Chem. Commun.* **2012**, *48*, 380–382.
3. Ning, J.; Wei, J.; Huang, S.; Wang, F., Luo, X., Sun, C.; Chen, D.; Wei, R.; Sha, L.; Liu, Y. A high performance Pb (ii) electrochemical sensor based on spherical CuS nanoparticle anchored gC_3N_4. *Anal. Methods* **2021**, *13*, 5617–5627.
4. Jahan, M.; Liu, Z.; Loh, K.P. A Graphene oxide and copper-centered metal organic framework composite as a tri-functional catalyst for HER, OER, and ORR. *Adv. Funct. Mater.* **2013**, *23*, 5363–5372.
